# How much evidence is needed before additional testing is incentivised at scale?

**DOI:** 10.3399/BJGP.2026.0357

**Published:** 2026-10-01

**Authors:** Martha MC Elwenspoek, Tracey Stone, Jonathan Decker, Penny Whiting, Jessica Watson

**Affiliations:** 1 Population Health Sciences, Bristol Medical School, University of Bristol, Bristol, UK

A recent update to a UK pay-for-performance scheme has introduced routine annual lipid testing for people with type 2 diabetes in primary care (Quality and Outcomes Framework [QOF] 2026/2027, indicator DM037). This does not align well with current guidelines and is based on limited evidence, yet it has immediate implications for clinician workload, patient experience, and the use of healthcare resources. It also highlights a broader challenge: how to ensure that financially incentivised care remains evidence-based and does not inadvertently promote low-value care.

Monitoring tests for long-term conditions account for a substantial proportion of primary care activity, representing around half of all testing.^1^ These tests are intended to assess disease progression, treatment response and adherence to therapy, and to detect complications or associated conditions such as cardiovascular disease. However, there is little robust evidence to guide which tests should be performed or how frequently.^2^ Existing guidance is largely based on expert opinion,^2–6^ and testing rates vary considerably between GP practices,^7^ reflecting ongoing clinical uncertainty.

This uncertainty carries risks. Crucially, the costs and harms of testing are not limited to the initial investigation itself. Unnecessary testing can lead to patient harm through anxiety, borderline results, and cascades of further investigations, including follow-up appointments, invasive tests, and referrals.^8–10^ More than half of abnormal results in primary care may be false positives,^11^ contributing to overdiagnosis and unnecessary treatment.^12^ At the same time, overtesting places additional strain on clinicians and healthcare resources, potentially diverting attention from interventions that offer clearer benefit. Without a stronger evidence base, there is a real risk some monitoring in long-term conditions provides limited patient benefit while increasing cost and harm.

Pay-for-performance schemes are widely used to improve quality of care in primary care. Financial incentives can be effective at standardising care and reducing variation,^13^ but they may also embed low-value or weakly evidenced practices into routine care, diverting resources from potentially more effective interventions. As illustrated by the example of annual lipid monitoring in type 2 diabetes, a key challenge is that incentivised processes do not always align with current guidelines or the best available evidence. De-implementation of unnecessary practices is difficult even when they are wasteful or potentially harmful.^14^ There should, therefore, be a high threshold for incentivising additional procedures at scale.

When direct evidence is lacking, alternative approaches are needed to guide practice. Ideally, randomised controlled trials would compare different monitoring strategies, but trials of sufficient duration to capture outcomes are often not feasible. To address this, we developed a methodology for evidence-based testing strategies using a series of filtering questions (Table 1), informed by rapid reviews, to identify tests unlikely to provide patient benefit. These criteria were applied to a list of commonly used tests for three long-term conditions: type 2 diabetes, hypertension, and chronic kidney disease, with final decisions informed by a consensus group of clinicians and patients.^15–17^

**Table 1.** Framework for assessing the value of monitoring tests

Testing rationale	Filtering questions
Monitoring disease progression	Can you intervene to slow or prevent further progression? Does earlier intervention improve outcomes, for example, reduce hospital admissions?
Monitoring therapy response	Can you intervene to improve treatment response? Does earlier intervention improve outcomes, for example, reduce hospital admissions?
Screen for other conditions	Is there an increased risk of this condition for people with the long-term condition of interest? Is the condition treatable? Is there a clear benefit to earlier detection or treatment?
Screen for adverse treatment effects	Is there an increased risk of this adverse treatment effect for people with the long-term condition of interest? Is the adverse effect treatable? Is there a clear benefit to earlier detection or treatment?

Applying these criteria to lipid testing in type 2 diabetes highlights the limitations of routine annual monitoring. Lipids are used to assess cardiovascular risk and guide statin prescribing, and there is strong evidence that lipid-lowering treatment reduces cardiovascular risk. However, cardiovascular risk can be assessed without lipid measurements, and statins are already recommended for most people with type 2 diabetes regardless of lipid levels. Current UK guidance reflects this, recommending testing at diagnosis and after treatment initiation, but not routine ongoing monitoring. Using our framework, the consensus group concluded that repeat lipid testing beyond these points offers little additional clinical value.

*“The challenges of overuse and low-value care have prompted international initiatives such as Choosing Wisely,* *as well as broader approaches such as prudent health care in Wales*, *and realistic medicine in Scotland.* *"*

In contrast, the recent QOF change appears to be based primarily on observational evidence linking missed completion of the diabetes care bundle to increased non-COVID-19 mortality.^18^ This evidence does not isolate the effect of lipid testing and is likely confounded by factors such as health-seeking behaviour, multimorbidity, and access to care. Introducing annual lipid testing on this basis risks increasing workload for clinicians and burden for patients without clear evidence of benefit. Applying structured criteria, such as those outlined here, could help ensure that future changes to incentivised care are based on a clear rationale and the best available evidence.

Monitoring tests may have potential benefits beyond direct clinical decision making, including supporting conversations between clinicians and patients and encouraging self-management. However, when we addressed this in a rapid review, we found mixed, limited, and low-quality evidence that additional testing improves lifestyle behaviours or patient outcomes.^15–17^ Testing may also have unintended consequences if results are misinterpreted, for example, by providing reassurance that further changes are unnecessary or reducing confidence in recommended treatments. As with potential harms, potential benefits of monitoring should therefore be demonstrated rather than assumed.

This raises a broader question: why are we less rigorous when it comes to monitoring than screening? Some monitoring tests directly assess disease progression, such as HbA1c in diabetes or estimated glomerular filtration rate in chronic kidney disease. However, many are effectively forms of targeted screening, aiming to detect related conditions in higher-risk populations, such as thyroid dysfunction in people with diabetes. Yet, while national screening programmes are subject to stringent criteria before implementation, monitoring practices often are not. The UK National Screening Committee applies clear criteria relating to the condition, the test, and the intervention to ensure that screening is more likely to benefit than harm. Given that much of routine monitoring in long-term conditions serves a similar purpose, applying comparable standards would seem both logical and necessary.

*“Before new monitoring tests are introduced at scale, there should be a clear evidence-based rationale demonstrating that the expected patient benefits outweigh the potential harms and resource implications.”*

National screening programmes offer a useful comparison. Many involve repeated testing, but the interval is explicitly justified based on the natural history of disease, test performance, and the balance of benefit and harm. In contrast, monitoring in long-term conditions is often repeated without the same level of scrutiny. Similar to screening programmes, the frequency of monitoring should be driven by clinical need and underlying biology, rather than by the structure of incentive frameworks such as QOF, which typically operate on an annual cycle.

The challenges of overuse and low-value care have prompted international initiatives such as Choosing Wisely,^19^ as well as broader approaches such as prudent health care in Wales^20^ and realistic medicine in Scotland.^21^ These initiatives recognise that more testing is not always better and emphasise evidence-based, personalised care. Similar principles should be embedded within payment incentive schemes. Before new monitoring tests are introduced at scale, there should be a clear evidence-based rationale demonstrating that the expected patient benefits outweigh the potential harms and resource implications. Financial incentives have an important role in reducing unwarranted variation in care, but they should only promote tests and care processes that meet these standards and align with clinical guidance. Incentive schemes should also allow flexibility, including longer retesting intervals and individualised decisions for patients unlikely to benefit, such as frail older adults or those with multimorbidity, so that testing remains guided by clinical judgement and patient need rather than performance targets.
